# Epigallocatechin Gallate and Glutathione Attenuate Aflatoxin B_1_-Induced Acute Liver Injury in Ducklings via Mitochondria-Mediated Apoptosis and the Nrf2 Signalling Pathway

**DOI:** 10.3390/toxins14120876

**Published:** 2022-12-15

**Authors:** Yanan Wang, Jiayu Wu, Lingfeng Wang, Ping Yang, Zuhong Liu, Shahid Ali Rajput, Mubashar Hassan, Desheng Qi

**Affiliations:** 1 Department of Animal Nutrition and Feed Science, College of Animal Science and Technology, Huazhong Agricultural University, Wuhan 430070, China; 2 Institute of Animal Husbandry and Veterinary Sciences, Wuhan Academy of Agricultural Sciences, Wuhan 430208, China; 3 Department of Animal Feed and Production, Faculty of Veterinary and Animal Sciences, Muhammad Nawaz Shareef University of Agriculture, Multan 66000, Pakistan

**Keywords:** aflatoxin B_1_, duckling, epigallocatechin gallate, glutathione, oxidative stress, Keap1/Nrf2 signalling, apoptosis

## Abstract

Aflatoxin B_1_ (AFB_1_) exists widely in feed and food with severe hazards, posing a serious threat to human and animal health. Epigallocatechin gallate (EGCG) and glutathione (GSH) have been reported as having anti-oxidative and other functions. The present study aimed to investigate the detoxification effect of EGCG and GSH alone or in combination on AFB_1_ exposure in ducklings. Fifty one-day-old male ducklings were randomly assigned into five experimental groups (*n* = 10): 1. Control (CTR); 2. 0.3 mg/kg BW AFB_1_ (AFB_1_); 3. 0.3 mg/kg BW AFB_1_ + 100 mg/kg BW EGCG (AFB_1_ + EGCG); 4. 0.3 mg/kg BW AFB_1_ + 30 mg/kg BW GSH (AFB_1_ + GSH); 5. 0.3 mg/kg BW AFB_1_ + 100 mg/kg BW EGCG + 30 mg/kg BW GSH (AFB_1_ + EGCG + GSH). The experiment lasted for seven days. Compared with the CTR group, AFB_1_ reduced growth performance, total serum protein and albumin content, increased serum enzyme activity (alanine aminotransferase, aspartate aminotransferase, alkaline phosphatase, and γ-glutamyl transpeptidase), and caused pathological damage to the ducklings’ livers. AFB_1_ exposure increased malondialdehyde content and decreased superoxide dismutase, total antioxidant capacity, catalase, glutathione peroxidase activities, and glutathione content in the liver. EGCG and GSH alone or in combination mitigated these adverse effects. Meanwhile, EGCG and GSH attenuate apoptosis of hepatocytes, and regulated AFB_1_-induced changes in the abundance of genes contained in the Keap1/Nrf2 signalling and apoptotic pathways. Collectively, these results suggest that EGCG and GSH alleviate the hepatocyte injury induced by AFB_1_ by inhibiting oxidative stress and attenuating excessive mitochondria-mediated apoptosis.

## 1. Introduction

Mycotoxins are harmful naturally occurring secondary metabolites produced by fungi [1]. Aflatoxins (AFs) are poisonous mycotoxins produced principally by *Aspergillus flavus* and *Aspergillus parasiticus*, of which Aflatoxin B_1_ (AFB_1_) is the most hepatotoxic [2]. Corn, wheat, and other grains have a high detection rate of AFB_1_ [3], which seriously affects the health of poultry and humans throughout the food chain [4]. AFB_1_ has been reported to cause diarrhoea, poor feather quality, weight loss, multifocal hepatic necrosis, bile duct hyperplasia, skeletal deformation, and altered muscle alignment in poultry [5,6]. Liver cancer, immunosuppression, and stunted children have all been linked with foods contaminated with AFB_1_ [7]. Over 70% of the world’s ducks are raised in China [8]. As ducklings are more sensitive to AFB_1_ than turkeys, lower doses of AFB_1_ can cause them bodily damage [9]. AFB_1_ exhibits its toxic action by being metabolised to exo-8, 9-epoxide (AFBO) in the liver [10]. The Keap1 (Kelch-like ECH-associated protein 1)/Nrf2 (nuclear factor erythroid 2-related factor 2) pathway enables cells to adapt to oxidative stress caused by external stimuli, and some plant extracts may activate this pathway [11]. Apoptosis plays an essential role in maintaining stability in the internal environment [12]. AFB_1_ can dysregulate the Keap1/Nrf2 pathway and cause excessive apoptosis in hepatocytes [13]. Hence, it will be an effective measure to improve the antioxidant capacity and inhibit excessive apoptosis caused by liver injury.

Epigallocatechin gallate (EGCG) is the main active ingredient in green tea. Other catechins include epicatechin-3-gallate, epigallocatechin and epicatechin, but EGCG is the most abundant [14]. It not only promotes animal growth performance and egg quality, ameliorates body fatty acid metabolism, and regulates intestinal health [15,16], but also contributes to cardioprotection, renoprotection, hepatoprotection, and neuroprotection in humans [17]. More importantly, EGCG is a scavenger of reactive oxygen/nitrogen and has potent antioxidant capacities. Moreover, it reduces the damage to cells caused by oxidative stress by capturing oxygen free radicals, restoring their redox status and mitochondrial function [18,19]. Previous studies have shown that EGCG can attenuate bleomycin-induced pulmonary fibrosis through the Keap1/Nrf2 signalling pathway [20]. Four hundred mg/kg of EGCG in the diet significantly alleviated heat stress in quail [21]. The preventive effect of EGCG against AFB_1_-induced liver injury and the mechanisms involved are not clarified. Therefore, it is necessary to proceed with this work.

Glutathione (GSH) is a tripeptide comprising cysteine, glutamic acid, and glycine. It exists in two forms in animals, oxidised (GSSG) and reduced (GSH), both of which play a significant role in bodily redox status [22]. The leading absorption site of exogenous GSH is the small intestine. Oral GSH in animals and humans can increase the content of GSH in the body [23], improve antioxidant capacity (i.e., protect cells from oxidative damage), protect intestinal mucosa, and enhance the transport and absorption of nutrients [22]. Studies have shown that GSH can increase the resistance of carp to nitric oxide stress and lipopolysaccharide (LPS) stimulation [24]. Adding GSH to the diet alleviated the oxidative damage caused by ochratoxin A and significantly inhibited cell apoptosis in rats [25]. Therefore, GSH may exhibit a positively beneficial effect in mitigating the hazards of AFB_1_.

The present study used EGCG and GSH to alleviate the damage caused by AFB_1_ in ducklings. In particular, we investigated whether EGCG and GSH could alleviate liver damage through the Keap1/Nrf2 signalling pathway and the inhibition of apoptosis and whether there was a mutual effect between them. The present experiment hoped to prompt the individual and combined use of EGCG and GSH to ameliorate toxin damage in animals.

## 2. Results

### 2.1. EGCG and GSH Inhibit AFB_1_-Induced Changes in Growth Performance and Liver Index of Ducklings

**Figure 1:**
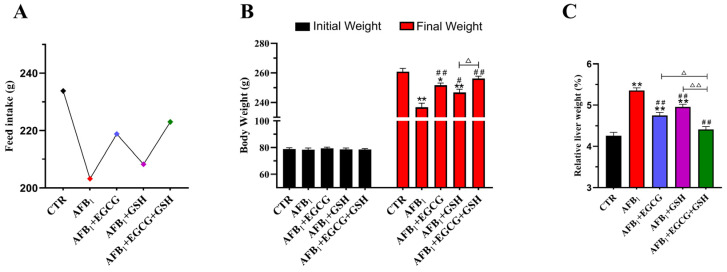
Effect of Epigallocatechin gallate (EGCG) and Glutathione (GSH) on Aflatoxin B_1_ (AFB_1_)-induced changes in growth performance and liver index of ducklings. (**A**) Average total feed intake per duckling during the experiment. (**B**) Body weight of each duckling at the beginning and end of the experiment. (**C**) Relative weight of the liver. Results are expressed as means ± SEM (*n* = 10). * *p* < 0.05, ** *p* < 0.01 vs. control (CTR) group; # *p* < 0.05, ## *p* < 0.01 vs. AFB_1_ group; ∆ *p* < 0.05, ∆∆ *p* < 0.01 significant difference between AFB_1_ + EGCG + GSH and AFB_1_ + EGCG or AFB_1_ + GSH groups.

The effects of AFB_1_ and EGCG or GSH on ducklings’ growth are shown in Figure 1A,B. In this experiment, ducks were treated with gavage, and each group of ten ducks was individually numbered but housed in a combined pen, so only the mean values of feed intake were calculated. As can be seen from the Figure 1A,B, there was no significant difference in the initial body weight, but after acute attacks, the AFB_1_ group had significantly reduced body weight (*p* < 0.01) compared with the CTR group, and the feed intake decreased by 13.1%. However, the EGCG and GSH alone and in combination significantly increased the ducklings’ body weights compared with the AFB_1_ group, while the feed intake increased by 7.7%, 2.5%, and 9.8%, respectively, showing that the combination of EGCG and GSH was more effective. As Figure 1C shows, AFB_1_ significantly increased the relative weight of the ducklings’ livers (*p* < 0.01), while EGCG and GSH significantly decreased as compared with the AFB_1_ group. The results indicate that EGCG and GSH alleviated the damage caused by AFB_1_, but a combination was more effective.

### 2.2. EGCG and GSH Protect against AFB_1_-Induced Liver Damage in Ducklings

**Figure 2:**
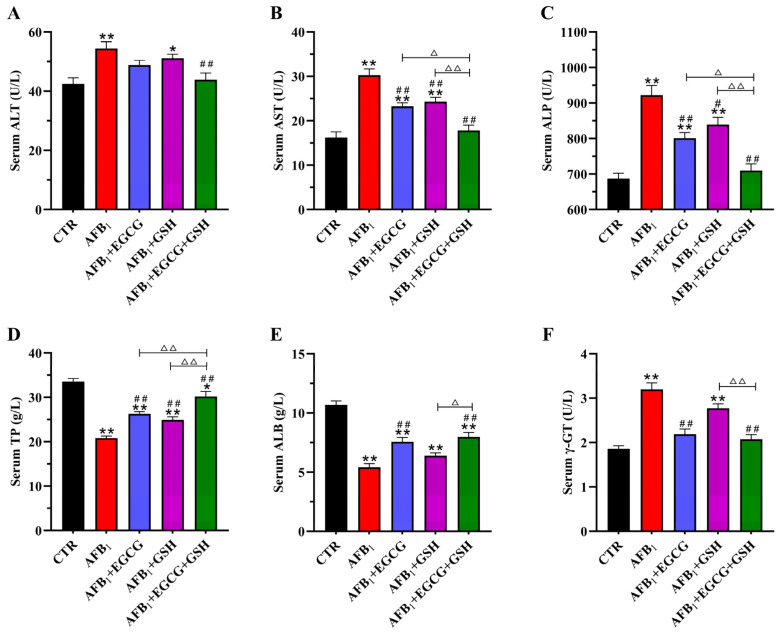
Effect of EGCG and GSH on AFB_1_-induced changes in serum biochemical parameters. (**A**) ALT, alanine aminotransferase; (**B**) AST, aspartate aminotransferase; (**C**) ALP, alkaline phosphatase; (**D**) TP, total protein; (**E**) ALB, albumin; (**F**) γ-GT, γ-glutamyl transpeptidase. Results are expressed as means ± SEM (*n* = 10). * *p* < 0.05, ** *p* < 0.01 vs. CTR group; # *p* < 0.05, ## *p* < 0.01 vs. AFB_1_ group; ∆ *p* < 0.05, ∆∆ *p* < 0.01 significant difference between AFB_1_ + EGCG + GSH and AFB_1_ + EGCG or AFB_1_ + GSH groups.

The effects of EGCG and GSH alone or in combination on the serum biochemistry of AFB_1_-exposed ducklings are shown in Figure 2. Serum biochemistry was affected adversely by AFB_1_ as the enzyme activities of alanine aminotransferase (ALT), aspartate aminotransferase (AST), alkaline phosphatase (ALP), and γ-glutamyl transpeptidase (γ-GT) were elevated (*p* < 0.01, Figure 2A–C,F). The use of EGCG or GSH mitigated these adverse effects. Compared with the AFB_1_ group, the enzyme activities of ALT, AST, ALP, and γ-GT decreased by 19.4%, 41.2%, 23.0%, and 35.2%, respectively, in the combined detoxification group. The AFB_1_-treated group reduced total serum protein (TP, 37.9%) and albumin (ALB, 49.2%) levels extremely significantly (*p* < 0.01; Figure 2D,E). Both EGCG or GSH increased the levels of TP and ALB compared with the AFB_1_ group, with the combined group increasing by 45.0% and 47.1%, respectively. This was still lower than the control group. The results indicate that EGCG and GSH alleviated the negative effects caused by AFB_1_ on the serum biochemistry of ducklings, but a combination was more effective.

### 2.3. EGCG and GSH Mitigate AFB_1_-Induced Oxidative Stress in the Livers of Ducklings

**Figure 3:**
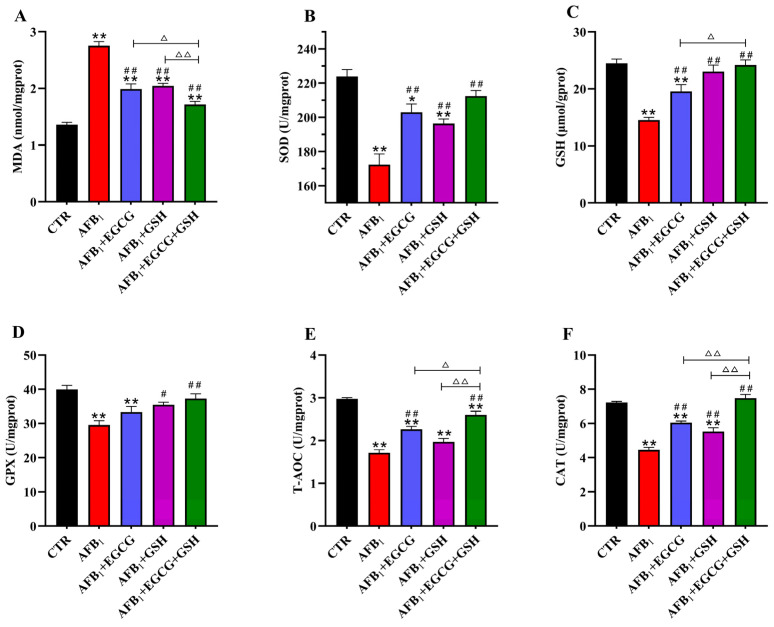
Effect of EGCG and GSH on AFB_1_-induced oxidative stress in the livers of ducklings. (**A**) MDA, malondialdehyde; (**B**) SOD, superoxide dismutase; (**C**) GSH, glutathione; (**D**) GPX, glutathione peroxidase; (**E**) T-AOC, total antioxidant capacity; (**F**) CAT, catalase. Results are expressed as means ± SEM (*n* = 10). * *p* < 0.05, ** *p* < 0.01 vs. CTR group; # *p* < 0.05, ## *p* < 0.01 vs. AFB_1_ group; ∆ *p* < 0.05, ∆∆ *p* < 0.01 significant difference between AFB_1_ + EGCG + GSH and AFB_1_ + EGCG or AFB_1_ + GSH groups.

To evaluate the damage caused by AFB_1_ and the protective effect of EGCG and GSH, we examined the antioxidant capacity of the ducklings’ livers. Compared with the CTR group, the AFB_1_ group highly significantly elevated malondialdehyde (MDA) content (*p* < 0.01), while EGCG, GSH, and a combination of both decreased MDA content by 27.8%, 25.7%, and 37.7%, respectively (Figure 3A). Meanwhile, the levels of antioxidant enzymes and GSH were also negatively affected, with the enzymatic activities of superoxide dismutase (SOD), glutathione peroxide (GPX), total antioxidant capacity (T-AOC), and catalase (CAT) decreasing by 23.0%, 26.0%, 34.2%, and 38.4% (Figure 3B,D–F), respectively, compared with the CTR group, while the levels of GSH decreased by 40.6% (Figure 3C). Thus, EGCG and GSH effectively prevented their alteration, especially in MDA, T-AOC, and CAT, where there was a significant joint effect (*p* < 0.05). The results indicate that EGCG or GSH alleviated the oxidative damage caused by AFB_1_, but a combination was more effective.

### 2.4. EGCG and GSH Prevent AFB_1_-Induced Alterations in the Microstructure and Ultrastructure of Duckling Livers

**Figure 4:**
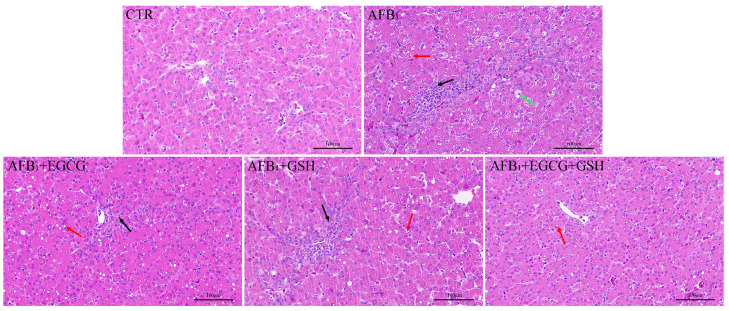
Effect of EGCG and GSH on the microscopic pathological structure of the livers of ducklings exposed to AFB_1_. Magnification 200×, scale bars = 100 µm. Green arrows: cell swelling and necrosis, nuclear pyknosis; black arrows: inflammatory cell infiltration in the hepatic parenchyma; red arrows: a small number of lipid droplets can be seen in the cytoplasm.

The liver is the target organ of AFB_1_ action, so we observed its microscopic and ultrastructural structure. As Figure 4 shows, the liver tissue structure of the CTR group was normal, with an intact hepatocyte structure and no fatty degeneration, necrosis, or inflammatory cell infiltration. However, we observed that large areas of hepatocytes were ill-defined, some cells were swollen and necrotic, and disappeared nuclei or pyknosis was present in the AFB_1_ group. Compared with the AFB_1_ group, inflammatory cell infiltration and hepatocyte necrosis were reduced in the detoxification group alone or in combination, especially in the combined detoxification group. However, lipid droplets were still present in some hepatocytes.

**Figure 5:**
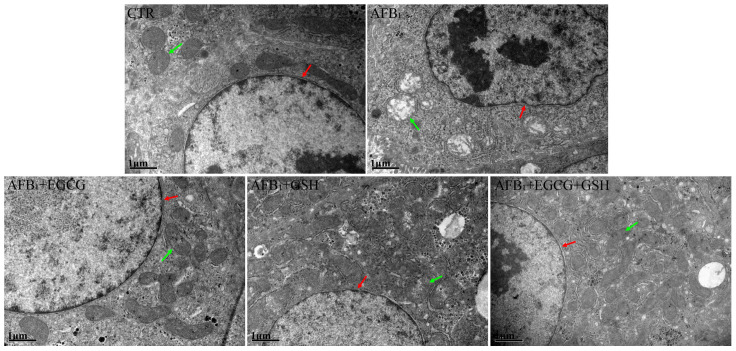
Effect of EGCG and GSH on the ultramicro-pathological structure of the livers of ducklings exposed to AFB_1_. Magnification 20,000×, scale bars = 1 µm. Red arrows represent the nucleus of the cell, and green arrows represent mitochondria.

To examine the internal structure of hepatocytes more closely, we performed transmission electron microscopy scans (Figure 5). In the CTR group, the nuclei and mitochondrial structures were normal, while in the AFB_1_ group, the nuclei underwent significant wrinkling, and the mitochondrial structures were heavily abnormal, with swelling and disrupted mitochondrial ridges. Although the mitochondrial structure was also lesioned to varying degrees in the EGCG or GSH groups, it was largely improved relative to the AFB_1_ group. The best results were seen in the combined group.

### 2.5. EGCG and GSH Alleviate the Interference of AFB_1_ on the Keap1-Nrf2 Antioxidant Signalling Pathway

**Figure 6:**
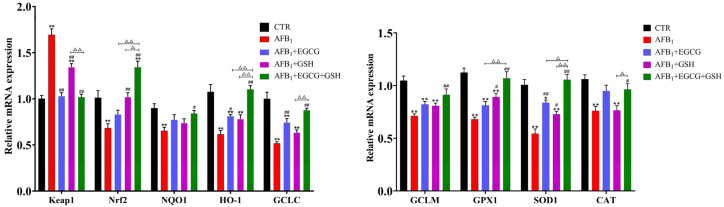
Effect of EGCG and GSH on the abundance of genes involved in the Keap1-Nrf2 signalling pathway in the livers of ducklings exposed to AFB_1_. All results are expressed as means ± SEM (*n* = 6). * *p* < 0.05, ** *p* < 0.01 vs. CTR group; # *p* < 0.05, ## *p* < 0.01 vs. AFB_1_ group; ∆ *p* < 0.05, ∆∆ *p* < 0.01 significant difference between AFB_1_ + EGCG + GSH and AFB_1_ + EGCG or AFB_1_ + GSH groups.

As Figure 6 shows, the abundance of related genes in the Nrf2 signalling pathway was significantly downregulated in the AFB_1_ group compared with the CTR group (*p* < 0.01). However, the gene expression of Keap1, an Nrf2 repressor, was significantly elevated (*p* < 0.01). These changes were back-regulated to varying degrees in the EGCG and GSH groups and in the combined group. The combined group Nrf2, HO-1 and SOD1 gene expression reached significant levels (*p* < 0.05) compared to the group used alone. The results indicate that EGCG and GSH can alleviate the oxidative damage caused by AFB_1_ and that they interact to some degree.

### 2.6. Protective Effects of EGCG and GSH on AFB_1_-Induced Apoptosis of Duckling Hepatocytes

**Figure 7:**
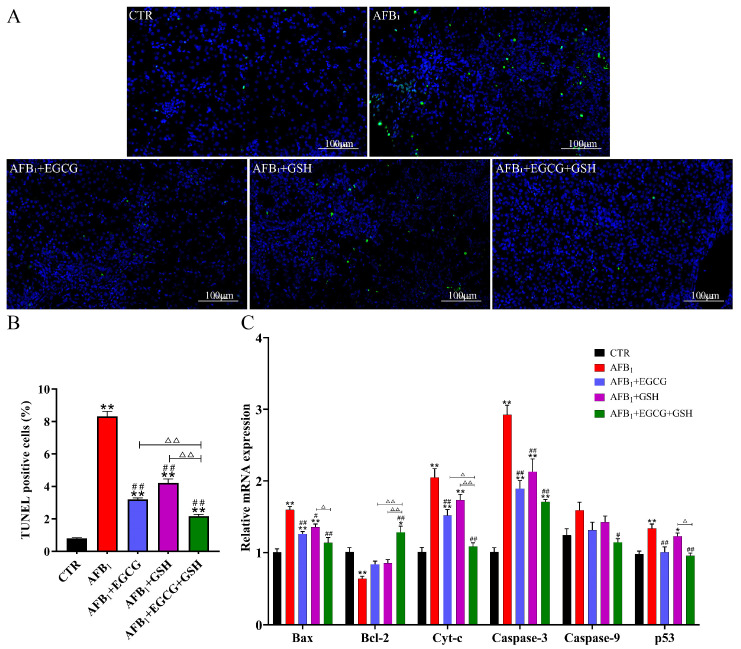
Effects of EGCG and GSH on AFB_1_-induced apoptosis of ducklings’ hepatocytes. (**A**) Terminal deoxynucleotidyl transferase dUTP nick end-labelling (TUNEL) staining to detect apoptosis. Magnification 200 ×, scale bars = 100 µm. Fluorescently labelled green indicates apoptotic cells, and blue indicates the nucleus. (**B**) TUNEL positive cells. (**C**) Expression of genes associated with mitochondria-mediated apoptosis, Cyt-c, Bax, Bcl-2, Caspase-3, Caspase-9, p53. All results are expressed as means ± SEM (*n* = 6). * *p* < 0.05, ** *p* < 0.01 vs. CTR group; # *p* < 0.05, ## *p* < 0.01 vs. AFB_1_ group; ∆ *p* < 0.05, ∆∆ *p* < 0.01 significant difference between AFB_1_ + EGCG + GSH and AFB_1_ + EGCG or AFB_1_ + GSH groups.

In order to evaluate the protective effect of EGCG and GSH alone or in combination against AFB_1_-induced apoptosis, hepatocyte apoptosis and the expression of genes related to apoptosis mediated by mitochondria were examined by terminal deoxynucleotidyl transferase dUTP nick end-labelling (TUNEL) staining and RT-qPCR, respectively. Green fluorescence was significantly enhanced (as evidenced in the increased number of apoptotic cells) in all AFB_1_-exposed groups (Figure 7A); the apoptosis rate (TUNEL positive rate) was elevated to 8.31% in the AFB_1_ group, while the apoptosis rate decreased by 61.5%, 49.2%, and 74.0% in the EGCG, GSH, and combined groups, respectively, compared with the AFB_1_ group (Figure 7B). Compared with the CTR group, the gene abundance of the pro-apoptotic gene Bax, as well as Cyt-c, Caspase-3, and p53 were significantly up-regulated (*p* < 0.01), and the anti-apoptotic gene Bcl-2 was significantly down-regulated (*p* < 0.01, Figure 7C) in the AFB_1_ group; EGCG and GSH alone or in combination had a positive effect. It was concluded that EGCG and GSH attenuated AFB_1_-induced apoptosis in hepatocytes.

## 3. Discussion

It is well-known that AFB_1_ is commonly found in feed and causes severe damage to commercial animals, especially ducks [26,27]. Growth retardation and hepatic lesion are among the most important symptoms of AFB_1_ poisoning. In the present study, we discovered that AFB_1_ reduced the ducklings’ feed intake and body weight, as well as caused liver damage. Our findings are consistent with previous research showing that AFB_1_ causes a decrease in food intake, metabolic capacity, body weight, and significantly higher liver coefficients [28,29,30]. The results of the present study indicated that EGCG and GSH significantly increased body weight and decreased liver indices. Serum ALT, AST, and ALP are the most sensitive indicators for evaluating liver damage, and AFB_1_ in the diet can increase the levels of these enzymes [31]. In one study, when ducklings were fed a diet of 0.1 mg/kg AFB_1_, the levels of AST, ALT, and the ratio of AST/ALT increased [32]. Because AFB_1_ inhibits protein biosynthesis, serum TB and ALB can be used to evaluate its impact [33]. Our results confirmed this: AFB_1_ elevated the levels of ALT, AST, ALP, and γ-GT while decreasing the content of TB and ALB compared with the CTR group. The addition of EGCG and GSH slowed down the change.

Oxidative stress can promote the formation of reactive oxygen species (ROS) in animal target organs [34]. Numerous studies have shown that excessive ROS can damage macromolecules such as proteins and nucleic acids, thereby producing a large amount of MDA. Therefore, MDA, as the end product of lipid peroxidation, is an important indicator for detecting oxidative damage [35,36]. However, excessive ROS in the body can be scavenged by antioxidant enzymes, including SOD, GPX, CAT, and GSH. In the present study, exposure to AFB_1_ increased the amount of MDA, while the content of GSH and the enzymatic activities of T-AOC, SOD, CAT, and GPX decreased. Apparently, AFB_1_ induced oxidative stress in the ducklings’ livers. The addition of EGCG and GSH also alleviated oxidative stress. Previous studies have shown that EGCG can attenuate carbon tetrachloride-induced oxidative stress in mouse livers and protect against H_2_O_2_-induced cellular oxidative damage [37,38]. Exogenous GSH has been found to have similar antioxidant effects in acute kidney injury in rats [39]. At the same time, we observed that AFB_1_ induced pathological changes in the liver. These results indicated that AFB_1_ caused oxidative damage to the liver, but EGCG and GSH could protect it by enhancing its antioxidant status. The antioxidant properties of EGCG and GSH themselves, as well as the fact that GSH can act as a substrate for GPX and GST, may explain the common effect exhibited by them.

Oxidative stress caused by AFB_1_ can regulate the expression of a series of genes involved in the antioxidant system through the Keap1-Nrf2 signalling pathway. Nrf2 is the main regulator of cells that respond to environmental stress, inducing the expression of detoxification and antioxidant enzymes. The activity of Nrf2 is dependent on the regulation of the Keap1 adaptor protein, which is a negative regulator of the former [40,41]. Under non-stress conditions, Nrf2 binds to Keap1 in the cytoplasm to promote Nrf2 ubiquitination and proteasomal degradation; when stimulated, Nrf2 dissociates from Keap1 into the nucleus and combines with nuclear receptors to regulate the expression of downstream target genes (NQO1, HO-1, GCLC, GCLM, and so on) [42], thereby performing antioxidant or detoxification functions. It has been demonstrated that EGCG can strengthen cellular defences against chemical carcinogens as well as ultraviolet (UV) and oxidative stress through the Keap1-Nrf2 signalling pathway [43]. In one experiment, the EGCG treatment group normalised the expression of Keap1-Nrf2 and its downstream regulatory proteins in fluoride-treated rat kidneys [44]. We noted a significant upregulation of Keap1 mRNA expression in the AFB_1_-treated group compared with the CTR group, indicating an enhanced negative regulation of Nrf2 by Keap1, while Nrf2 and its related genes (NQO1, HO-1, GCLC, GCLM, SOD1, GPX1, and CAT) were downregulated. Treatment with EGCG significantly reversed these effects (a finding that is consistent with previous studies). However, the effect of GSH on this pathway has not been investigated, so we speculated that it might regulate the expression of genes by balancing ROS production. This deserves more investigation. We concluded that EGCG and GSH contribute to the antioxidant capacity of the body through the Keap1-Nrf2 signalling pathway and that they are most effective in combination.

Apoptosis (i.e., programmed cell death) plays an essential role in controlling cell numbers and maintaining the homeostasis of multicellular organisms. Abnormal regulation of apoptosis has been associated with the development of a variety of diseases [45]. AFB_1_ has been reported to induce apoptosis in hepatic, pulmonary, and bone marrow cells [46,47]. The present study found hepatocytes undergoing significant apoptosis in the AFB_1_ group. It is widely known that mitochondria perform a central role in apoptosis initiated by many kinds of stimuli and that key events in apoptosis are associated with mitochondria [48]. Livers have been observed with severe mitochondrial lesions. In such cases, membrane permeability is altered, and Cyt-c enters the cytoplasm from the mitochondria, binding to the apoptosis protease activator Apaf-1 and caspase-9 and activating caspase-9, which in turn induces the activation of caspase-3 and subsequently triggers apoptosis mediated by the mitochondria [49]. However, mitochondria-mediated apoptosis is regulated by the pro-apoptotic protein Bax and the anti-apoptotic protein Bcl-2 [50]. The present study showed that AFB_1_ reduced the mRNA expression of Bcl-2 and elevated the mRNA expression of Bax, while the expression of associated apoptotic genes (Cyt-c, caspase-3, caspase-9, and so on) was significantly elevated. However, EGCG and GSH inhibited the excessive apoptosis of hepatocytes caused by AFB_1_ by regulating the expression of these genes. Studies have shown that EGCG protects against apoptosis in human umbilical vein endothelial cells by regulating the mitochondria-dependent apoptotic signalling pathway [51], while exogenous GSH defends IPEC-J2 cells from oxidative stress-induced apoptosis [52]. Apoptosis can be activated by oxidative stress [36]. Therefore, we speculate that EGCG and GSH alleviate apoptosis caused by AFB_1_ in hepatocytes either directly or by inhibiting oxidative stress. However, the mechanism of interaction between EGCG and GSH needs to be further researched.

## 4. Conclusions

In the present study, AFB_1_ caused serious damage to the ducklings. The results suggest that EGCG and GSH can alleviate acute liver injury by improving hepatic antioxidant capacity through the Keap1-Nrf2 signalling pathway and inhibiting the excessive apoptosis of hepatocytes mediated by mitochondria. This explains the protective mechanism of EGCG and GSH alone or in combination against AFB_1_-induced liver injury. The present study also provides a theoretical basis for their application, and we suggest that EGCG and GSH could be used as promising duck feed additives to counteract AFB_1_ damage.

## 5. Materials and Methods

### 5.1. Animals and Experimental Design

Age is an important factor affecting the bird’s resistance to AFB_1_, and male ducklings are more sensitive (male ducklings produce more AFBO than females), so we chose younger males to complete the experiment [53]. One-day-old male Cherry Valley ducks were purchased from Wuhan Yongsheng Duck Industry Co., Ltd. (Wuhan, China). The ducklings were kept in a controlled environment at a temperature of 30 ± 2 °C and 60 ± 5% humidity. The experiment (No. HZAUDU-2022-0002) was approved by the Animal Ethics Committee of Huazhong Agricultural University (Wuhan, China).

After three days of acclimatisation, 50 male ducklings were randomly divided into five groups (*n* = 10): 1. Control group (CTR); 2. Treated with AFB_1_ (>99%, Pribolab, Qingdao, China) 0.3 mg/kg BW (AFB_1_); 3. Treated with AFB_1_ 0.3 mg/kg BW + EGCG (98%, Shanghai Yuanye Biotech Co., Ltd., Shanghai, China) 100 mg/kg BW (AFB_1_ + EGCG); 4. Treated with AFB_1_ 0.3 mg/kg BW + GSH (Reduced, 98%, Aladdin, Shanghai, China) 30 mg/kg BW (AFB_1_ + GSH); 5. Treated with AFB_1_ 0.3 mg/kg BW + EGCG 100 mg/kg BW + GSH 30 mg/kg BW (AFB_1_ + EGCG + GSH). Each group of ten ducklings was kept in a pen, marked and weighed individually. All ducklings were gavaged with the same concentration and 1 mL Volume/200 g BW. The acute liver injury experiment cycle lasted for 7 days. On Days 1–6, they were weighed daily and gavaged with distilled water, distilled water, EGCG, GSH, and EGCG + GSH, respectively. On Day 4, Groups 2 to 5 were treated with AFB_1_ 0.5 h after the first gavage, and the control group was given the corresponding solvent gavage (4% dimethyl sulfoxide). Slaughter sampling took place on Day 7. The composition and nutrient levels of the basal diet are shown in Appendix A, Table A1. The acute toxic dose of AFB_1_ was determined based on previous reports [54,55] and preliminary experiments. Gavage doses of EGCG and GSH refer to preliminary experiment. The health status of the ducklings was strictly observed, and the body weight and feed intake were recorded during the experiment.

### 5.2. Sample Collection

After the ducklings fasted for 12 h, blood samples were collected using wing venipuncture into a tube. The blood samples were centrifuged at 3500 rpm for 10 min to obtain serum, which was divided and stored at −80 °C for biochemical analysis. The ducklings were immediately sacrificed and dissected to remove the liver, rinsed in cold saline, and weighed. A portion of liver tissue was cut and placed in paraformaldehyde fixative and 2.5% glutaraldehyde, respectively, for hematoxylin and eosin (H&E) staining or TUNEL detection and ultrastructural observation. The remaining part of each liver was stored at −80 °C in a refrigerator to detect antioxidant indexes, gene expression, and so on. The relative weight of the livers was calculated using the following formula:

Relative weight = liver weight (g)/body weight (g) × 100%

### 5.3. Determination of Serum Biochemical Indicators

The serum enzyme activities of ALT, AST, ALP, and γ-GT, as well as the content of ALB and TP, reflect the function of the liver. These indicators were measured using an automatic biochemical analyser according to the manufacturer’s set procedure (Mindray, Shenzhen, China). The serum samples were placed in a cryogenic sample tray. ALT, AST, ALP, and γ-GT were expressed as U/L, while ALB and TP were expressed as g/L. All of these kits were purchased from the same manufacturer (Mindray, Shenzhen, China).

### 5.4. Detection of Antioxidant Capacity of the Liver

Liver tissue homogenates were prepared according to the requirements of the corresponding kits, and the protein concentration of the homogenate supernatant was determined by the BCA protein assay kit (Beyotime Biotechnology, Shanghai, China). SOD, GPX, MDA, T-AOC, CAT, and GSH kits were procured and operated according to the manufacturer’s instructions (Nanjing Jiancheng Biotech, Nanjing, China). Absorbance was measured by a microplate reader (Multiskan MK3, Thermo Fisher Scientific, Waltham MA, USA) or visible light spectrophotometer (722E, Shanghai Spectrum Instruments Co., Ltd., Shanghai, China), and the enzyme activity or substance content was calculated and analysed.

### 5.5. Histopathological Analysis

Fresh liver tissue samples were placed in 4% paraformaldehyde and fixed for more than 24 h. The tissues were dehydrated with different concentrations of alcohol and embedded in wax. The wax blocks were placed in a microtome (Leica RM2016, Wetzlar, Germany) and cut into sections of 4 μm thickness. Staining with hematoxylin and eosin was performed for histopathological observation.

### 5.6. Ultrastructural Pathology Observation

Liver samples were cut to around 1 mm^3^ in size and placed in 2.5% glutaraldehyde for 24 h. After 24 h, the samples were washed three times with 0.1 M PBS and fixed with 1% osmium acid for 2 h. The samples were rewashed with 0.1 M PBS, then dehydrated with gradient acetone and embedded in resin. The embedded samples were cut into 60 nm sections using an ultramicrotome (Leica UC5, Wetzlar, Germany), stained with uranyl acetate and lead citrate solution, and observed under a transmission electron microscope (Hitachi H-7650, Tokyo, Japan) for scanning and photographing [56].

### 5.7. Detection of Apoptosis by TUNEL Staining

Following the TUNEL kit manufacturer’s instructions (Roche, Basel, Switzerland), the embedded liver sections were dewaxed, rehydrated, and then incubated with proteinase K at 37 °C for 30 min and washed three times with PBS. Fifty 50 μL of TUNEL reaction solution were added dropwise to the tissue, incubated at 37 °C for 2 h in the dark, washed again with PBS three times, and then incubated with 4,6-diamidino-2-phenylindole (DAPI) staining solution for 10 min while keeping it out of the light. After blocking, the images were observed and collected using an inverted fluorescence microscope (Olympus IX51, Tokyo, Japan). Fluorescence signals were analysed with Image-Pro Plus 6.0 (Media Cybernetics, Silver Spring, MD, USA), and apoptosis rates were calculated.

### 5.8. Quantitative Real-Time PCR

Total RNA was extracted from the ducklings’ livers using Trizol reagent (TaKaRa, Dalian, China), and the quality (A260/A280) and concentration were evaluated using a NanoDrop 2000 spectrophotometer (Thermo Fisher Scientific, Waltham, MA, USA). Genomic DNA was removed, and RNA was reverse transcribed into cDNA using PrimeScript^TM^ RT reagent Kit with gDNA Eraser (TaKaRa, Dalian, China) according to the steps in the instructions, and expressed genes were evaluated by Real-Time PCR using TB Green^®^ Premix Ex Taq^TM^ II (TaKaRa, Dalian, China). The primer sequences are shown in Appendix A Table A2. All primers were designed by Sangon Biotech (Shanghai, China) and synthesised by Tsingke Biotechnology Co., Ltd. (Beijing, China). The relative mRNA abundance was analysed following the 2^−∆∆Ct^ formula and normalised with the housekeeping gene GAPDH [57].

### 5.9. Statistical Analysis

The results were analysed using one-way ANOVA with SPSS Version 26 (SPSS Incorporated, Armonk, NY, USA) and Tukey’s multiple comparisons as the post-hoc test. Outcomes were expressed as mean ± standard error (SEM). GraphPad Prism Version 9.0 (GraphPad Prism, San Diego, CA, USA) was used to visualise the data. In all statistical analyses, *p* < 0.05 was considered significant and *p* < 0.01 was considered highly significant.

## Data Availability

The data presented in this study are available on request from the corresponding author.

## Appendix A

**Table A1:** Composition and nutrient level of basal diet.

Ingredients	Content (%)	Nutrition Component ^2^	Content (%)
Corn	52.98	Crude protein	20.16
Soybean meal (44% CP)	30.36	ME (MJ/kg)	12.34
Fish meal	2.50	Calcium	0.91
Wheat bran	3.00	Available phosphorus	0.42
Rice bran	5.25	Methionine	0.45
Soybean oil	1.91	Lysine	1.12
Premix ^1^	4.00	AFB_1_ (μg/kg)	2.1
Total	100.00		

^1^ Premix provided per kilogram of diet: 10,000 IU of vitamin A, 2500 IU of vitamin D_3_, 35 IU of vitamin E, 2.5 mg of vitamin K_3_, 2.5 mg of vitamin B_1_, 9 mg of vitamin B_2_, 0.02 mg of vitamin B_12_, 15 mg of calcium pantothenate, 60 mg of niacin, 1.5 mg of folic acid, and 0.2 mg of biotin; 12 mg of Cu, 80 mg of Fe, 60 mg of Zn, 92 mg of Mn, 0.3 mg of Se, and 0.3 mg of I. ^2^ All nutrient levels were calculated.

**Table A2:** Primer sequences used in qRT-PCR.

Genes	Primer Sequences (5′ to 3′)	Product Lengths (bp)	Accession No.
Keap1	F: TCAAGACCTCACCCTCCATAAACCC R: AGTAGCCCAAGGACTGCCGATAG	110	KU048807.1
Nrf2	F: TGGCTGAGGTAAGCACAATCACAAG R: GGCTCTCAACAGTCTCCAGGAAATC	138	NM_001310777.1
NQO1	F: TGTCACCATCTCCGACCTCTATGC R: TCCTTCCACGCTTCTCCCATCTC	126	XM_027466610.2
HO-1	F: ATGAATGCCCTTGAGATGGACCTTG R: GTGACCGTTCTCCTGGCTCTTTG	132	XM_005015345.5
GCLC	F: TTCAGGTGACATTCCAGGCTTGC R: AGAACGGAGATGCAGCACTCAATG	108	XM_027455103.2
GCLM	F: TGTTGTGTGATGCCACCTGATCTC R: GTGCTTTGACGTTCTGGATGCTTTC	142	XM_027462629.2
SOD1	F: TCATCTCTCTGACTGGACCACACTG R: GTTAGCGTGCTCTCGTTGTCTCC	103	KU048808.1
GPX1	F: CCACCAGGAGAATGCCACCAAC R: CTTCCCGTTCACCTCGCACTTC	115	XM_027467953.2
CAT	F: ATGGACCAATGTGCGTGACTGAC R: CATGCGGCTCTCCTTCACAACAG	104	XM_027458335.2
Cyt-c	F: CCAGTGCCATACGGTTGAGAAGG R: TCTGTGTAGGAGAAGCCCTCAGC	105	XM_027447873.2
Bax	F: TCGTCGCCCTCTTCTACTTCGC R: CAGGAGACGATGGTGCGGAAAAG	88	KY788660.1
Bcl-2	F: CATGTGCGTGGAGAGCGTCAAC R: ACTGATCCAGCCTCCGTTGTCC	124	XM_027451679.2
Caspase-3	F: TGAGGCAGACAGTGGACCAGATG R: TCCTTCAGCACCCTACACAGAGAC	156	XM_021279218.3
Caspase-9	F: TGGATTGCGATTCACCCGAAGATG R: ATTACCCGAGGGAGCCTGGAAAG	83	XM_038166520.1
p53	F: AGGAGGAGAACTTCCGCAAGAGG R: GCAGGCAGAAGATCTCGTTGTCG	129	XM_038171818.1
GAPDH	F: GTCTCCTGCGACTTCAACGG R: CCTTGGATGCCATGTGGACC	160	XM_038180584.1
